# Omalizumab for the reduction of allergic reactions to foods: a narrative review

**DOI:** 10.3389/falgy.2024.1409342

**Published:** 2024-05-28

**Authors:** Hafsa Ghouri, Ashna Habib, Zainab Nazir, Nimerta Lohana, Aymar Akilimali

**Affiliations:** ^1^ Department of Medicine, Dow University of Health Sciences, Karachi, Pakistan; ^2^ Department of Medicine, Liaquat University of Medical and Health Sciences, Jamshoro, Pakistan; ^3^ Medical Research Circle, Goma, Democratic Republic of Congo

**Keywords:** Omalizumab, Xolair, Omalizumab (Xolair), allergic reaction, food allergy, IgE

## Abstract

The frequency of food allergies varies between 2% and 10%, depending on characteristics including age, region, race, and method of diagnosis self-reported by patients or oral food challenges (OFCs). The most common allergies reported are tree nuts (1.2%), milk (1.9%), peanuts (2.2%), and shellfish (1.3%). Omalizumab injection has now been approved by the FDA for the treatment of immunoglobulin E-mediated food allergies in specific adults and children aged one year or older. This medication reduces the risk of allergic reactions (Type I), which can include anaphylaxis, when an individual accidentally encounters one or more food allergens. Omalizumab functions by binding to IgE and altering IgE-mediated pathways, which lessens IgE's capacity to cause allergic reactions. Promising outcomes from clinical trials and case studies include lowered anaphylactic risk and enhanced tolerance to allergens. Omalizumab, however, may have adverse effects; thus, close observation is required. Overall, this review sheds light on the efficacy, safety, and clinical implications of omalizumab, highlighting its potential as a useful intervention for IgE-mediated food allergies.

## Introduction

Food-related allergies, which are defined as adverse immune-mediated reactions to food proteins, are becoming more common (1). It needs to be distinguished from non-immune-mediated adverse food reactions, such as toxic (like food poisoning), pharmacologic (like caffeine), and metabolic (like lactose intolerance) events (2). Food allergy (FA) is categorized as immunoglobulin E (IgE)-mediated, non-IgE-mediated, or mixed based on the kind of immunological response (3). IgE-mediated food allergies are type I hypersensitivity reactions that happen when an individual develops IgE antibodies against a food protein and then is exposed to that protein (1). The typical symptoms of food allergies can impact various bodily systems, including the skin, respiratory tract, gastrointestinal tract, cardiovascular system, and nervous system. Skin-related symptoms like rashes, itching, hives, and swelling are particularly common (4). The prevalence of food allergies ranges from 2% to 10%, influenced by factors such as age, geographic location, racial background, and how the allergy is diagnosed—whether through oral food challenges (OFCs) or self-reported by patients (1). The most frequently reported allergens include peanuts (2.2%), milk (1.9%), shellfish (1.3%), and tree nuts (1.2%) (5). Double-blind placebo-controlled OFCs are the gold standard diagnostic method for FA (DBPCFC). To minimize the danger of deadly anaphylaxis associated with OFC, further complementary diagnostic procedures ought to be carried out prior to the challenge test. The risk of OFC may be determined with the use of a thorough clinical history, skin prick test (SPT), sIgE level, and component-resolved diagnostic (CRD) testing (6). Currently, oral immunotherapy (OIT) is advised for children ages 4–5 who have a chronic cow milk, hen's egg, or peanut allergy due to its capacity to elevate the threshold for adverse reactions (7). The recommendation is supported by strong data from a meta-analysis that demonstrated the efficacy of oral immunotherapy for children with allergies to peanuts, hen eggs, and cow milk (8). A humanized antibody called Omalizumab can bind free IgE, decrease cell-bound IgE, and lower high-affinity Fc*ε*RI receptors. This results in a reduction in mediator release, which in turn reduces allergic reactions (9). The first and only non-antihistamine medication approved for the medical management of chronic spontaneous utricaria (CSU) is omalizumab. It is authorized for the treatment of CSU that is resistant to antihistamines in patients who are 12 years of age or older. Its efficacy is evident from the data (including RCTs) compiled in several systematic reviews and meta-analyses (10–12). Additionally, it is the first biological therapy licensed for IgE-mediated persistent allergic asthma (13). Omalizumab's effectiveness and tolerability in treating severe asthma in subjects with tIgE values >1,500 IU/ml and comorbidities such as IgE-mediated food allergies and allergic rhinitis were assessed in a case series involving seven patients aged 7–18 years. Omalizumab was successful in reducing asthma symptoms as measured by the ACT score and in raising spirometry readings after two years of medication; no patient had asthma exacerbations or required ER visits. Furthermore, during the oral food challenge test (OFC), all individuals with food allergies successfully managed to develop “desensitization” to the triggering food (14).

Omalizumab injection has now been approved by the FDA for the treatment of immunoglobulin E-mediated food allergies in specific adults and children aged one year or older. This medication reduces the risk of allergic reactions (Type I), which can include anaphylaxis, when an individual accidentally encounters one or more food allergens (15). The goal of this review is to thoroughly assess omalizumab's possible contribution to the management of IgE-mediated food allergies as well as the reduction of allergic reactions to food. This review aims to provide insights into the efficacy, safety, and practical aspects of omalizumab therapy for food allergies by combining existing information from clinical trials, case studies, and real-world experiences.

## Methodology

This narrative review analyzes the relevant literature, including clinical studies on Omalizumab and its use to reduce allergic reactions to food. A search of the literature was done from the beginning to February 10, 2024, using Google Scholar, PubMed, and Clinical Trials databases. “Omalizumab,” “Food Allergy,” “Xolair,” and other relevant keywords were among the search terms used. English-language articles were included. Additionally, outside resources like the NHS, CDC, WHO, and others were consulted for specific information. This review does not include abstract-only articles, commentaries, letters to the editor, or papers written in languages other than English.

## Understanding allergic reactions to foods

IgE-mediated food allergies develop as an outcome of a breakdown in the critical immune system mechanisms that sustain tolerance while avoiding harmless food antigens from being identified as pathogens (5, 16). IgEs attach themselves to mast cell surface receptors. The adverse effects of IgE-mediated allergic reactions are caused by the patient being exposed to the same food antigen again, which binds specific IgE bound to Fc*ε*R on the surfaces of mast cells and basophil cells, causing those cells to degranulate and release mediators like histamine (17). The past ten years have seen an increase in the prevalence of food allergies, making this a significant public health concern. False diagnoses can be reduced if doctors are aware of the limits of using clinical history alone to detect food allergies and use testing carefully. Improvements in therapeutics, prevention measures, and diagnostics are desperately needed, considering the growing incidence of food allergies. To develop more effective interventions for both the treatment of current food allergies and their prevention, ongoing efforts are being made to get a better understanding of the mechanisms causing and sustaining food allergies, as well as how these mechanisms differ among individuals.

## Mechanistic insights into omalizumab

**Figure 1:**
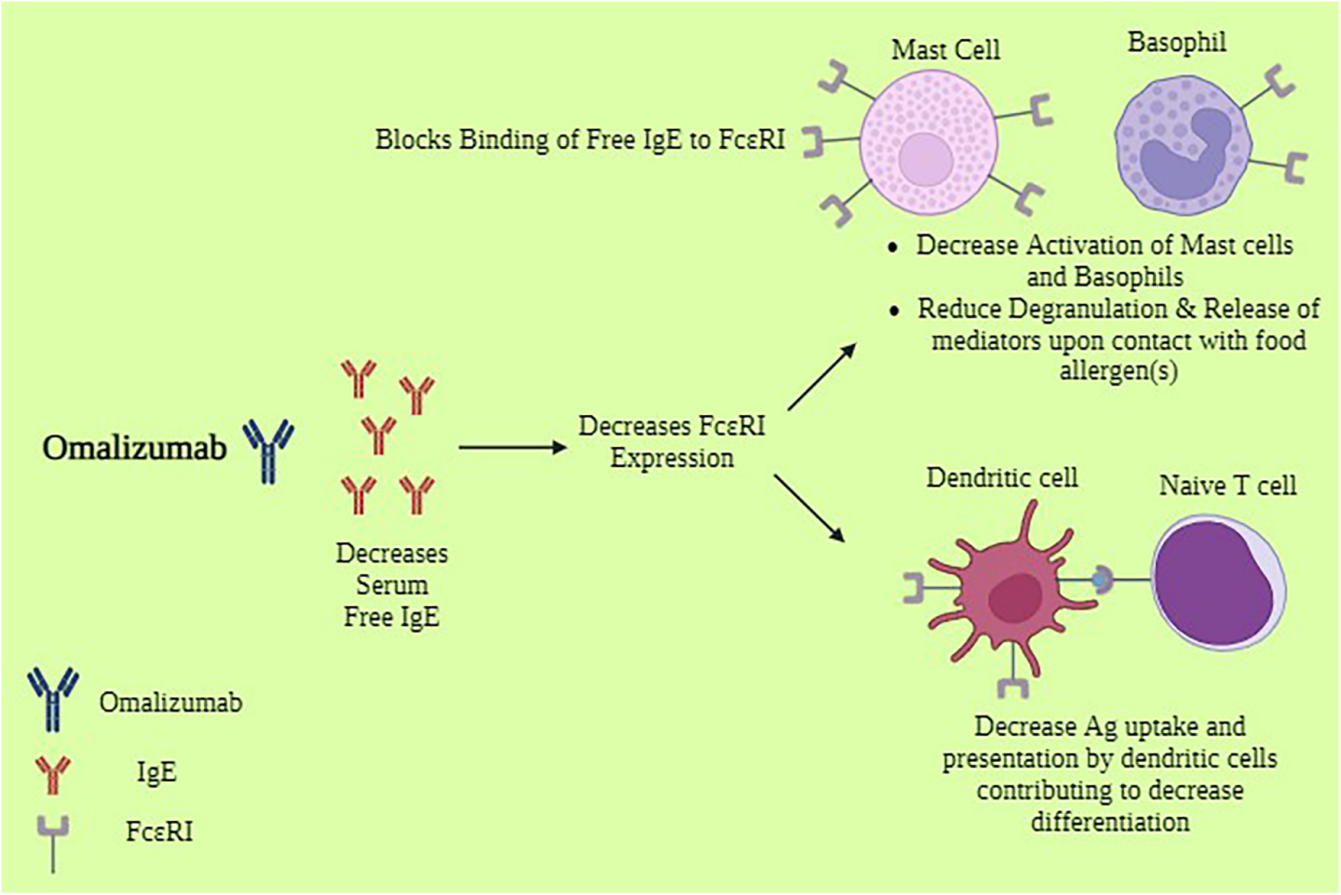
Mechanism of action of omalizumab*. *Biorender. Available online at: https://app.biorender.com (Assessed May 12, 2024).

Omalizumab acts by binding free IgE, which significantly lowers the quantity of IgE that is accessible to bind antigen on the surface of mast cells and basophils. Data indicate a 98% decrease in free blood IgE levels in patients receiving omalizumab (18). It's interesting to note, though, that a decrease in blood levels of free IgE does not always correspond with improved clinical results with omalizumab medication (19). This implies that omalizumab's additional immunomodulatory actions might have practical significance. IgE can control how its own high-affinity receptor (Fc*ε*RI) is expressed on the cell surface (20). Increased numbers of IgE receptors expressed on mast cells and basophils have been correlated with higher IgE levels. Omalizumab can break this positive feedback loop by reducing free IgE levels. It has been demonstrated to do so on a variety of cell types, such as basophils and mast cells (both cutaneous and lung) (18, 21–24) as shown in Figure 1. As a result, these cells are less likely to degranulate in reaction to the allergen. Omalizumab not only inhibits the expression of Fc*ε*RI on basophils and mast cells, but it can also cause a functional alteration in these cells by reducing the release of histamine when antigen is encountered (18). This result was further supported by research involving patients receiving omalizumab treatment for allergies, asthma, and peanuts (25–27). In contrast to mast cell histamine release, which happened only later in therapy, one study examining nasal allergic responses in patients with cat allergies revealed that clinical outcomes related to reductions in basophil histamine release (BHR) (26). Table 1 demonstrates the potential benefits of Omalizumab in food allergy management.

**Table 1:** Potential benefits of omalizumab in food allergy management.

Potential benefits	Description
Reduction of IgE Levels	By binding to circulating IgE, omalizumab reduces its ability to cause allergic reactions (28), which could reduce the severity of reactions to allergens in food.
Decreased risk of anaphylaxis	Omalizumab could reduce the chance of experiencing severe allergic reactions, such as anaphylaxis, when exposed to food allergens by reducing free IgE levels.
Increased tolerance threshold	Treatment with omalizumab has been linked to an increase in the threshold level for allergy reactions (29), enhancing patient safety and lowering concern around unintentional exposures.
Improved desensitization outcomes	When used in conjunction with food allergy immunotherapy (AIT), omalizumab can improve the safety and effectiveness of the treatment, lowering severe adverse events and raising tolerance to allergens (30).
Extended durability of desensitization	Omalizumab has the potential to maintain the desensitized state over time and decrease allergy responses following successive exposures by extending the durability of the desensitization attained with AIT.
Improved quality of life	Effective omalizumab treatment can improve quality of life by minimizing the fear and stress of severe allergic reactions and allowing for more dietary choice and social participation.
Potential for early intervention	Omalizumab provides an early intervention option for newborns and young children with severe food allergies, with the potential to prevent the development of comorbid diseases and improve long-term results.

## Targeting IgE and allergic pathways

The Food and Drug Administration (FDA) originally approved omalizumab, a humanized anti-IgE monoclonal antibody, to treat severe allergic asthma (31). Later, it was also licensed to treat refractory chronic spontaneous urticaria (32) and CRSwNP (33–35). It was originally developed to prevent free IgE from attaching to high-affinity receptors on effector cells, such as basophils and mast cells, but it may also make it easier for inflammatory complexes to separate from one another. Omalizumab is the sole FDA-approved anti-IgE biopharmaceutical that targets and inhibits IgE. Off-label use of omalizumab for allergens, including AR, has also been shown (36).Omalizumab decreases B cell-producing IgE by inducing anergy in membrane IgE-bearing B cells (37), eosinophil apoptosis (22), and a decrease in serum eosinophil count. Interestingly, an *in vitro* study using omalizumab on human tonsillar B cells revealed a decrease in the number of IgE + B cells. This is likely because the drug targets membrane-bearing B cells in human B cells, reducing their ability to synthesize IgE (37).

## Clinical trials and studies

Leung DY et al. studied anti-IgE as a treatment for food allergies for the first time in 2003 (38). In their randomized, double-blind, placebo-controlled study, an anti-IgE molecule called TNX-901 was evaluated on peanut-allergic patients. For four months, 84 patients between the ages of 12 and 60 received monthly subcutaneous injections of either TNX-901 or a placebo. To prevent unintentional peanut ingestion, the highest dose (450 mg) considerably raised the threshold dose of peanut required to cause a clinical reaction. The mean tolerated peanut dose increased in patients who took 450 mg of TNX-901, possibly offering protection in the event of accidental consumption. When compared to the placebo, all three of the drug's doses reduced serum-free IgE levels by 88% or more from baseline, and they were all well tolerated with comparable adverse effects (38). Following an agreement amongst pharmaceutical companies interested in the development of anti-IgE treatments, further research utilizing this antibody was terminated. Omalizumab's potential utility as a treatment for food allergies was examined in further studies. Sampson et al. started a phase II trial of omalizumab as a therapy for peanut allergies (39). According to the study's limited data, participants who got omalizumab were able to tolerate more peanuts than those who received a placebo at the post-24-week therapy peanut challenge (*P* = 0.054). Furthermore, after 24 weeks of therapy, 44% of the omalizumab-treated participants could tolerate the goal dose of 1 g of peanut protein, compared to only 1 subject in the placebo-treated group (39). In another trial, omalizumab treatment for 6 months was evaluated for symptomatic and cellular reactions in 14 patients with an allergy to peanuts (27). After 6 months of treatment, a significant reduction in skin prick test titration was noticed. Omalizumab resulted in a substantial rise in the threshold dosage for oral food challenge with peanuts, leading to allergic reactions (from 80 to 6,500 mg, *P* = 0.01). In addition, peanut-induced basophil release of histamine was completely inhibited in five patients and decreased by ten times in nine patients (27). Schneider et al. (40) conducted a study examining the use of omalizumab alongside an oral peanut desensitization protocol in high-risk peanut-allergic subjects. Thirteen pediatric patients, with a median age of 10 years (range 8–16 years), were involved. The patients underwent a 12-week omalizumab course (following European dosing guidelines) before starting the initial rush oral desensitization, which gradually increased up to 500 mg of peanut flour over 6 h. Over the subsequent 8 weeks, they continued with a slower weekly escalation up to a final maintenance dose of 4,000 mg of peanut flour, after which omalizumab was discontinued while daily oral peanut ingestion continued. A second double-blind, placebo-controlled food challenge was conducted 12 weeks after omalizumab cessation. Patients were followed for 52 weeks. Out of the 12 participants who completed the study, all 12 (100%) eventually passed an oral food challenge (OFC) with a cumulative dose of 8,000 mg of peanut flour. During the six-month observation period following omalizumab discontinuation and while on maintenance dosing, 17 reactions were recorded, two of which were severe, leading to four instances of epinephrine use at home in two subjects (40). Omalizumab, when combined with immunotherapy, has been shown to be both safe and effective in rapidly desensitizing patients. However, the effectiveness of different doses of Omalizumab in enabling immunotherapy remains a significant question that needs clarification. Ongoing research is investigating the therapeutic potential of various biologics that target cytokines like IL-4, IL-13, IL-5, and others, aiming to address this issue (41). In a phase II trial of 4–15-year-old children who had allergies to 2–5 foods, omalizumab improved their ability to successfully complete an oral food challenge to a minimum of 2-g food protein for two or more foods after 36 weeks of treatment (83% vs. 33% without omalizumab, *P* = .004) (42). Omalizumab additionally increased the safety of multi-oral immunotherapy by lowering the proportion of doses linked to adverse events (AEs) from 68% without omalizumab to 27% with omalizumab (*P* = .008) (42). High levels of oral and gastrointestinal side effects are reported in studies looking at oral immunotherapy (OIT) without omalizumab, and these effects may cause some patients to stop their medication (43, 44). Omalizumab may therefore be able to decrease these side effects and increase the number of people who are able to withstand desensitization procedures. In a recent analysis (45) of 177 children and adolescents aged 1–17 years, 79 out of 118 (67%) who received omalizumab successfully ingested a single dose of 600 mg or more of peanut protein without experiencing dose-limiting symptoms, compared to 7% of placebo-receiving ones (*P* < 0.001). An additional finding included ingesting cashew, milk, and egg in single doses of at least 1,000 mg each without dose-limiting symptoms; the result was consistent (cashew, 41% vs. 3%; milk, 66% vs. 10%; egg, 68% vs. 0%; *P* < 0.001 for all comparisons) (45).

## Adverse effects

Although omalizumab is essential for treating diseases, it can have several side effects that need to be monitored. The two most frequently reported side effects of omalizumab were injection site reactions and fever (45). The side effects might range from less common occurrences like skin blistering or reddening, soreness in the muscles or joints, and difficulty moving, to more significant consequences like tightness in the chest, an irregular heartbeat, or even cancerous tumors (46). While some adverse effects, including unusual fatigue or bleeding gums, might not be apparent right away, they still need to be treated by a doctor (46). Less serious side effects include body aches, chilly symptoms, dry tongue, and responses at the injection site that usually go away with time (46). However, if any adverse effects worsen or continue, it's crucial to speak with a medical professional.

## Case studies and clinical experience

The instances that follow highlight the wide range of patients and clinical settings in which omalizumab has been used, providing insight into the medication's effectiveness and safety profile. Nishie et al. reported on a 51-year-old lady who was treated with omalizumab for 6 years after developing oral steroid-dependent severe asthma (47). She suffered from an allergy to shellfish and wheat, as well as an oral allergy condition brought on by kiwis and other foods that are associated with latex-fruit syndrome. Her symptoms from a food allergy had vanished after starting the omalizumab medication. Disseminated erythema abruptly developed after 7 years of treatment; omalizumab was stopped due to a possible drug-induced eruption. She experienced worsening asthma control right after consuming wheat, along with a tickling feeling in her throat following the omalizumab interruption. Omalizumab was administered again, which alleviated these symptoms. This suggested that omalizumab may have caused the patient's food allergy to remit in addition to improving asthma management (47). Rocha et al. discussed two patients with various food allergies and eosinophilic esophagitis who were on a highly restrictive diet and were treated with omalizumab to ameliorate food intolerance, which was the most distressing factor in their lives (48). The patients' stated symptoms showed a marked improvement. On the other hand, histology and esophageal endoscopy did not show any improvement (48). Sakamoto et al. described the case of a 12-year-old kid who had been suffering from perennial allergic rhinitis since the age of eight (49). He began experiencing recurrent episodes of lip edema at the age of eleven after consuming raw fruits and vegetables. A diagnosis of pollen-food allergy syndrome (PFAS) was made for him. He was told to stay away from foods that cause problems. Administration of omalizumab reduced lip edema. Omalizumab and sublingual immunotherapy together may be a useful treatment option for PFAS patients, according to Sakamoto et al. (49). Nilsson et al. reported that omalizumab was administered to five children aged 6–16 with severe milk allergies, including bouts of anaphylaxis and IgE antibodies to casein and alpha-lactalbumin (milk proteins) ranging from 30 to 160 kUA/L (50). Before and after four months of Omalizumab, CD-sens was tested; if the results were negative, an oral milk challenge was administered. Every child had a negative milk challenge and a negative CD-sens; however, one child needed to take twice as much omalizumab to reach a negative CD-sens before a challenge could be completed. Nilsson et al. stated that when treating severe food allergies, such as milk anaphylaxis, omalizumab seems to be helpful, and CD-sens monitoring can help determine when and how to conduct a food challenge (50). Klein et al. presented a 22-year-old male with severe cow's milk allergy who had recurrent anaphylactic episodes since infancy and continued into maturity, with sometimes severe immediate-type reactions after accidental intake (51). The patient's medical history includes bronchial asthma under control. Omalizumab and cetirizine were started as off-label treatments. Following a prolonged course of treatment, the patient underwent a double-blind oral exposure test to cow's milk. Thus, 14 ml might be accepted. Following the ingestion of thirty milliliters of cow's milk, angioedema, dyspnea, and urticaria appeared. The patient's tolerance to cow's milk increased while receiving omalizumab therapy. The authors reported that reactions during inadvertent ingestion could therefore be avoided (51). Takahashi et al. presented the case of a 5-year-old boy with severe allergies who developed anaphylaxis after ingesting cow's milk (CM) (52). Prior to the open-injection therapy (OIT), his total immunoglobulin E (IgE) level was 654 IU/ml. In the skin prick test (SPT), he showed erythema and wheals. Following a 0.2 ml CM intake, he received 150 mg of omalizumab every other week for a total of 8 weeks. He underwent a rush phase of OIT before being released to resume his daily 200 ml CM dosage at home. After a year of omalizumab treatment, the end-point titration values from the SPT declined, despite higher sIgE levels, and the SPT was negative. Five months after daily CM intake stopped for two weeks, an oral meal challenge was conducted, and the omalizumab was resumed. During the expedited OIT, the patient only had five mild adverse events, and his quality of life significantly improved. The authors suggested that in these cases, a negative SPT could be helpful in guiding the stoppage of omalizumab (52). Suzuki et al. presented the case of a 30-year-old lady with refractory asthma (53). She had also eaten peaches and had twice had severe anaphylactic symptoms. The patient abstained from eating any fruit, including peaches, out of extreme caution that anaphylaxis would occur again. Due to her food allergy, she was instructed to strictly avoid eating peaches. Omalizumab was the first medication to be started to better regulate asthma and diet. In a brief period, the peak expiratory flow rose, and the asthmatic symptoms subsided. The consumption limit for peaches was progressively eased. This was started 28 weeks after the patient started omalizumab medication and was accomplished by challenging the patient with increasing dosages of 290 mg of peach fruit. The patient did not have any allergic reactions after eating peaches after the limitation on peach consumption was eventually lifted. Omalizumab treatment was therefore very successful in helping this patient achieve remission from both peach allergy and asthma (53). While omalizumab has shown promise as a therapeutic option for patients with severe allergic diseases, including food allergies, careful consideration of its advantages and hazards is required in clinical practice. These case studies advance our knowledge of omalizumab's possible use in the treatment of food allergies.

## Ongoing research and future directions

An unmet medical need for efficient therapies beyond allergen avoidance and emergency treatment was addressed by the FDA's approval of Omalizumab, which will reduce allergic reactions in some patients. This marks a significant improvement in the management of food allergies. The long-term safety and effectiveness of Omalizumab, as well as its possible uses in other allergic disorders, are still being studied. This research will advance our knowledge of immunotherapy techniques for the management of allergies. Even with Omalizumab's approval to treat IgE-mediated food allergies, there are still several unmet needs and room for growth in this field. Omalizumab has shown promise in lowering allergy reactions to certain food allergens, but it has drawbacks as well. These include the need for ongoing allergen avoidance and the possibility of side effects, which highlight the need for greater research into more all-encompassing treatment approaches. To address the various difficulties related to food allergies and enhance patient outcomes, future research could explore combination medicines, customized treatment plans, and innovative immunomodulatory drugs. To guarantee equal access to efficient allergy management techniques, it is also crucial to improve Omalizumab and comparable treatment accessibility, affordability, and patient education.

## Challenges and limitations

Omalizumab and its use in treating food allergies present several research challenges, one of which is the need for strong study designs and procedures to guarantee the validity and reliability of results. The intricacies associated with food allergies and the wide range of patient groups they impact require the use of strict procedures in clinical trials, such as suitable standards for patient selection, standardized outcome measurements, and sufficient sample sizes. In addition, it is vital to tackle possible partialities and confusing elements to precisely evaluate the security and effectiveness of Omalizumab, thereby assisting in the development of evidence-based clinical judgment. There are several difficulties and restrictions when implementing the data from Omalizumab clinical studies for IgE-mediated food allergies into standard clinical practice. When administering Omalizumab in real-world situations, healthcare practitioners may run into challenges with patient eligibility requirements, reimbursement considerations, and practical issues. The broad use of Omalizumab as a food allergy treatment option may also be hampered by the requirement for specific training in the management of allergic disorders and the organization of multidisciplinary care teams. To effectively promote the integration of evidence-based practices into clinical care pathways, it is imperative that stakeholders, including healthcare professionals, policymakers, and patient advocacy groups, collaborate to address these obstacles. Overcoming potential implementation and access constraints is critical to the successful use of Omalizumab treatment for IgE-mediated food allergies. Access to Omalizumab may be restricted by factors like cost, insurance coverage constraints, and gaps in the healthcare infrastructure, especially for underprivileged groups and those with limited financial resources. Moreover, logistical issues pertaining to medicine delivery, storage, and administration must be resolved to guarantee prompt and fair access to Omalizumab, particularly in isolated or resource-constrained environments. Advocating for the extension of insurance coverage, creating patient support programs, and establishing collaborative care models that enable the coordinated delivery of allergy management treatments across healthcare settings are some strategies to improve access and implementation. By taking proactive measures to remove these obstacles, interested parties can enhance the cost-effectiveness and availability of Omalizumab therapy for people with IgE-mediated food allergies, thereby enhancing patient outcomes and quality of life.

## Conclusion and recommendations

For eligible patients with IgE-mediated food allergies, especially those who are at risk of experiencing severe allergic responses, healthcare practitioners are recommended to think about Omalizumab as a therapeutic option. Recommendations for the proper administration of Omalizumab, such as dose schedules, monitoring procedures, and patient selection standards, should be included in clinical practice guidelines. Further investigation is required to clarify the long-term safety and effectiveness of Omalizumab, find treatment response predictors, and investigate cutting-edge therapy approaches to meet the unmet requirements of people with food allergies. In conclusion, Omalizumab's approval represents a major development in the treatment of food allergies mediated by IgE, providing hope to both patients and medical professionals. Even though Omalizumab cannot treat food allergies, its capacity to lower the likelihood of allergic reactions is a significant advancement in the quality of life for those who suffer from this medical condition. Through additional research into the function of omalizumab and other immunotherapies in the management of food allergies, we can work toward a time when everyone will have access to efficient treatments, ultimately lessening the negative effects of food allergies on health and improving the lives of those who are impacted.

## References

[1] NIAID-Sponsored Expert Panel B, Boyce JA Assa’ad A Burks AW Jones SM Sampson HA Guidelines for the diagnosis and management of food allergy in the United States: report of the NIAID-sponsored expert panel. J Allergy Clin Immunol. (2010) 126(6 Suppl):S1–58. 10.1016/j.jaci.2010.10.007 21134576 PMC4241964

[2] Moore LE Stewart PH deShazo RD. Food allergy: what we know now. Am J Med Sci. (2017) 353(4):353–66. 10.1016/j.amjms.2016.11.014 28317623

[3] Yu W Freeland DMH Nadeau KC. Food allergy: immune mechanisms, diagnosis and immunotherapy. Nat Rev Immunol. (2016) 16(12):751–65. 10.1038/nri.2016.111 27795547 PMC5123910

[4] Ho MHK Wong WHS Chang C. Clinical spectrum of food allergies: a comprehensive review. Clin Rev Allergy Immunol. (2014) 46(3):225–40. 10.1007/s12016-012-8339-6 23229594

[5] Gupta RS Warren CM Smith BM Blumenstock JA Jiang J Davis MM The public health impact of parent-reported childhood food allergies in the United States. Pediatrics. (2018) 142(6):1–12. 10.1542/peds.2018-1235 PMC6317772 30455345

[6] Sampson HA Gerth van Wijk R Bindslev-Jensen C Sicherer S Teuber SS Burks AW Standardizing double-blind, placebo-controlled oral food challenges: American academy of allergy, asthma & immunology–European academy of allergy and clinical immunology PRACTALL consensus report. J Allergy Clin Immunol. (2012) 130(6):1260–74. 10.1016/j.jaci.2012.10.017 23195525

[7] Pajno GB Fernandez-Rivas M Arasi S Roberts G Akdis CA Alvaro-Lozano M EAACI guidelines on allergen immunotherapy: IgE-mediated food allergy. Allergy. (2018) 73(4):799–815. 10.1111/all.13319 29205393

[8] Nurmatov U Dhami S Arasi S Pajno GB Fernandez-Rivas M Muraro A Allergen immunotherapy for IgE-mediated food allergy: a systematic review and meta-analysis. Allergy. (2017) 72(8):1133–47. 10.1111/all.13124 28058751

[9] Incorvaia C Mauro M Russello M Formigoni C Riario-Sforza GG Ridolo E. Omalizumab, an anti-immunoglobulin E antibody: state of the art. Drug Des Devel Ther. (2014) 8:197–207. 10.2147/DDDT.S49409 24532966 PMC3923619

[10] Bernstein JA Kavati A Tharp MD Ortiz B MacDonald K Denhaerynck K Effectiveness of omalizumab in adolescent and adult patients with chronic idiopathic/spontaneous urticaria: a systematic review of ‘real-world’ evidence. Expert Opin Biol Ther. (2018) 18(4):425–48. 10.1080/14712598.2018.1438406 29431518

[11] Urgert MC Elzen MT Knulst AC Fedorowicz Z Zuuren EJ. Omalizumab in patients with chronic spontaneous urticaria: a systematic review and GRADE assessment. Br J Dermatol. (2015) 173(2):404–15. 10.1111/bjd.13845 25891046

[12] Zhao ZT Ji CM Yu WJ Meng L Hawro T Wei JF Omalizumab for the treatment of chronic spontaneous urticaria: a meta-analysis of randomized clinical trials. J Allergy Clin Immunol. (2016) 137(6):1742–50.e4. 10.1016/j.jaci.2015.12.1342 27040372

[13] Xolair. European Medicines Agency [WWW Document] (n.d.). Available online at: https://www.ema.europa.eu/en/medicines/human/EPAR/xolair (cited May 13, 2024).

[14] Dinardo G Cafarotti A Galletta F Fiocchi A Arasi S. Omalizumab in severe asthma and food allergies with IgE levels >1500 kU/L: two-year evaluation. Pediatr Allergy Immunol. (2023) 34(12):1–5. 10.1111/pai.14057 38146110

[15] FDA. FDA Approves First Medication to Help Reduce Allergic Reactions to Multiple Foods After Accidental Exposure (2024). Available online at: https://www.fda.gov/news-events/press-announcements/fda-approves-first-medication-help-reduce-allergic-reactions-multiple-foods-after-accidental (cited March 17, 2024).

[16] Wambre E Bajzik V DeLong JH O’Brien K Nguyen QA Speake C A phenotypically and functionally distinct human TH2 cell subpopulation is associated with allergic disorders. Sci Transl Med. (2017) 9(401):1–21. 10.1126/scitranslmed.aam9171 PMC5987220 28768806

[17] Irani C Maalouly G. Prevalence of self-reported food allergy in Lebanon: a middle-eastern taste. Int Sch Res Notices. (2015) 2015:1–5. 10.1155/2015/639796 PMC4897070 27347535

[18] MacGlashan DW Bochner BS Adelman DC Jardieu PM Togias A McKenzie-White J Down-regulation of fc(epsilon)RI expression on human basophils during in vivo treatment of atopic patients with anti-IgE antibody. J Immunol. (1997) 158(3):1438–45. 10.4049/jimmunol.158.3.1438 9013989

[19] Korn S Haasler I Fliedner F Becher G Strohner P Staatz A Monitoring free serum IgE in severe asthma patients treated with omalizumab. Respir Med. (2012) 106(11):1494–500. 10.1016/j.rmed.2012.07.010 22884459

[20] MacGlashan DW Jr Bochner BS Adelman DC Jardieu PM Togias A Lichtenstein LM. Serum IgE level drives basophil and mast cell IgE receptor display. Int Arch Allergy Immunol. (1997) 113(1–3):45–7. 10.1159/000237504 9130480

[21] Beck LA Marcotte GV MacGlashan D Jr Togias A Saini S. Omalizumab-induced reductions in mast cell Fc*ε*RI expression and function. J Allergy Clin Immunol. (2004) 114(3):527–30. 10.1016/j.jaci.2004.06.032 15356552

[22] Djukanović R Wilson SJ Kraft M Jarjour NN Steel M Chung KF Effects of treatment with anti-immunoglobulin E antibody omalizumab on airway inflammation in allergic asthma. Am J Respir Crit Care Med. (2004) 170(6):583–93. 10.1164/rccm.200312-1651OC 15172898

[23] Lin H Boesel KM Griffith DT Prussin C Foster B Romero FA Omalizumab rapidly decreases nasal allergic response and Fc*ε*RI on basophils⋆. J Allergy Clin Immunol. (2004) 113(2):297–302. 10.1016/j.jaci.2003.11.044 14767445

[24] Gomez G Jogie-Brahim S Shima M Schwartz LB. Omalizumab reverses the phenotypic and functional effects of IgE-enhanced Fc*ε*RI on human skin mast cells. The Journal of Immunology. (2007) 179(2):1353–61. 10.4049/jimmunol.179.2.1353 17617628 PMC2396781

[25] Noga O Hanf G Kunkel G Kleine-Tebbe J. Basophil histamine release decreases during omalizumab therapy in allergic asthmatics. Int Arch Allergy Immunol. (2008) 146(1):66–70. 10.1159/000112504 18087163

[26] Eckman JA Sterba PM Kelly D Alexander V Liu MC Bochner BS Effects of omalizumab on basophil and mast cell responses using an intranasal cat allergen challenge. J Allergy Clin Immunol. (2010) 125(4):889–95.e7. 10.1016/j.jaci.2009.09.012 19962744 PMC2850969

[27] Savage JH Courneya JP Sterba PM MacGlashan DW Saini SS Wood RA. Kinetics of mast cell, basophil, and oral food challenge responses in omalizumab-treated adults with peanut allergy. J Allergy Clin Immunol. (2012) 130(5):1123–9.e2. 10.1016/j.jaci.2012.05.039 22800401 PMC3935509

[28] Lowe PJ Renard D. Omalizumab decreases IgE production in patients with allergic (IgE-mediated) asthma; PKPD analysis of a biomarker, total IgE. Br J Clin Pharmacol. (2011) 72(2):306–20. 10.1111/j.1365-2125.2011.03962.x 21392073 PMC3162660

[29] Mortz CG Parke L Rasmussen HM Kjaer HF Bindslev-Jensen C. A randomized, double-blind placebo-controlled study on the efficacy of omalizumab on food allergy threshold in children with severe food allergy. Allergy. (2024) 79(4):964–76. 10.1111/all.16046 38366983

[30] Zhang Y Zhang M Zhang J Li Q Lu M Cheng L. Combination of omalizumab with allergen immunotherapy versus immunotherapy alone for allergic diseases: a meta-analysis of randomized controlled trials. Int Forum Allergy Rhinol. (2024) 14(4):794–806. 10.1002/alr.23268 37715592

[31] Kotoulas SC Tsiouprou I Fouka E Pataka A Papakosta D Porpodis K. Omalizumab: an optimal choice for patients with severe allergic asthma. J Pers Med. (2022) 12(2):165. 10.3390/jpm12020165 35207654 PMC8878072

[32] Hide M Park HS Igarashi A Ye YM Kim TB Yagami A Efficacy and safety of omalizumab in Japanese and Korean patients with refractory chronic spontaneous urticaria. J Dermatol Sci. (2017) 87(1):70–8. 10.1016/j.jdermsci.2017.03.009 28366435

[33] Gevaert P Omachi TA Corren J Mullol J Han J Lee SE Efficacy and safety of omalizumab in nasal polyposis: 2 randomized phase 3 trials. J Allergy Clin Immunol. (2020) 146(3):595–605. 10.1016/j.jaci.2020.05.032 32524991

[34] Han JK Yoo B Saenz R Braid J Millette LA Lee SE. Omalizumab and quality of life in nasal polyps: a post hoc analysis. Int Forum Allergy Rhinol. (2022) 12(9):1188–90. 10.1002/alr.22963 34979056 PMC9545462

[35] Gevaert P Saenz R Corren J Han JK Mullol J Lee SE Long-term efficacy and safety of omalizumab for nasal polyposis in an open-label extension study. J Allergy Clin Immunol. (2022) 149(3):957–65.e3. 10.1016/j.jaci.2021.07.045 34530020

[36] El-Qutob D. Off-label uses of omalizumab. Clin Rev Allergy Immunol. (2016) 50(1):84–96. 10.1007/s12016-015-8490-y 26048266

[37] Chan MA Gigliotti NM Dotson AL Rosenwasser LJ. Omalizumab may decrease IgE synthesis by targeting membrane IgE+ human B cells. Clin Transl Allergy. (2013) 3(1):29. 10.1186/2045-7022-3-29 24004581 PMC3875359

[38] Leung DYM Sampson HA Yunginger JW Burks AW Schneider LC Wortel CH Effect of anti-IgE therapy in patients with peanut allergy. N Engl J Med. (2003) 348(11):986–93. 10.1056/NEJMoa022613 12637608

[39] Sampson HA Leung DYM Burks AW Lack G Bahna SL Jones SM A phase II, randomized, double-blind, parallel-group, placebo-controlled oral food challenge trial of Xolair (omalizumab) in peanut allergy. J Allergy Clin Immunol. (2011) 127(5):1309–10.e1. 10.1016/j.jaci.2011.01.051 21397314

[40] Schneider LC Rachid R LeBovidge J Blood E Mittal M Umetsu DT. A pilot study of omalizumab to facilitate rapid oral desensitization in high-risk peanut-allergic patients. J Allergy Clin Immunol. (2013) 132(6):1368–74. 10.1016/j.jaci.2013.09.046 24176117 PMC4405160

[41] Passanisi S Caminiti L Zirilli G Lombardo F Crisafulli G Aversa T Biologics in food allergy: up-to-date. Expert Opin Biol Ther. (2021) 21(9):1227–35. 10.1080/14712598.2021.1904888 33733975

[42] Andorf S Purington N Block WM Long AJ Tupa D Brittain E Anti-IgE treatment with oral immunotherapy in multifood allergic participants: a double-blind, randomised, controlled trial. Lancet Gastroenterol Hepatol. (2018) 3(2):85–94. 10.1016/S2468-1253(17)30392-8 29242014 PMC6944204

[43] Varshney P Jones SM Scurlock AM Perry TT Kemper A Steele P A randomized controlled study of peanut oral immunotherapy: clinical desensitization and modulation of the allergic response. J Allergy Clin Immunol. (2011) 127(3):654–60. 10.1016/j.jaci.2010.12.1111 21377034 PMC3060783

[44] Burks AW Jones SM Wood RA Fleischer DM Sicherer SH Lindblad RW Oral immunotherapy for treatment of egg allergy in children. N Engl J Med. (2012) 367(3):233–43. 10.1056/NEJMoa1200435 22808958 PMC3424505

[45] Wood RA Togias A Sicherer SH Shreffler WG Kim EH Jones SM Omalizumab for the treatment of multiple food allergies. N Engl J Med. (2024) 390(10):889–99. 10.1056/NEJMoa2312382 38407394 PMC11193494

[46] DrugsCom. Omalizumab Side Effects: Common, Severe, Long Term (n.d.). Available online at: https://www.drugs.com/sfx/omalizumab-side-effects.html (cited March 17, 2024).

[47] Nishie M Masaki K Okuzumi S Mochimaru T Kabata H Miyata J Successful treatment of a patient with adult food allergy and severe asthma using omalizumab. Asia Pac Allergy. (2021) 11(3):e27. 10.5415/apallergy.2021.11.e27 34386403 PMC8331258

[48] Rocha R Vitor AB Trindade E Lima R Tavares M Lopes J Omalizumab in the treatment of eosinophilic esophagitis and food allergy. Eur J Pediatr. (2011) 170(11):1471–4. 10.1007/s00431-011-1540-4 21809010

[49] Sakamoto D Hamada S Kobayashi Y Shimono M Shimamura A Kanda A Omalizumab is effective for a patient with pollen-food allergy syndrome who experienced intractable lip edema. Auris Nasus Larynx. (2023) 50(5):805–10. 10.1016/j.anl.2022.12.001 36581536

[50] Nilsson C Nordvall L Johansson SGO Nopp A. Successful management of severe cow’s milk allergy with omalizumab treatment and CD-sens monitoring. Asia Pac Allergy. (2014) 4(4):257–60. 10.5415/apallergy.2014.4.4.257 25379486 PMC4215434

[51] Klein B Simon JC Treudler R. Treatment of severe cow’s milk allergy with omalizumab in an adult. Allergol Select. (2023) 7:20–4. 10.5414/ALX02372E 36756389 PMC9904097

[52] Takahashi M Taniuchi S Soejima K Hatano Y Yamanouchi S Kaneko K. Successful desensitization in a boy with severe cow’s milk allergy by a combination therapy using omalizumab and rush oral immunotherapy. Allergy Asthma Clin Immunol. (2015) 11(1):18. 10.1186/s13223-015-0084-y 26064142 PMC4461908

[53] Suzuki S Matsuura T Kimura T Tazaki T Fukuda M Homma T A case of severe asthma and peach allergy that improved with omalizumab therapy: a case report. Arerugi. (2012) 61(2):215–23. 22437731

